# Changing the structure of PFOA and PFOS: a chemical industry strategy or a solution to avoid thyroid-disrupting effects?

**DOI:** 10.1007/s40618-024-02339-w

**Published:** 2024-03-24

**Authors:** F. Coperchini, A. Greco, M. Rotondi

**Affiliations:** 1 Department of Internal Medicine and Therapeutics, University of Pavia, Via S. Maugeri 4, 27100 Pavia, Italy; 2 Laboratory for Endocrine Disruptors, Unit of Endocrinology and Metabolism, Istituti Clinici Scientifici Maugeri IRCCS, 27100 Pavia, Italy

**Keywords:** PFOA, PFOS, Endocrine disruptor, Thyroid

## Abstract

**Background:**

The family of perfluoroalkyl and polyfluoroalkyl substances (PFAS) raised concern for their proven bioaccumulation and persistence in the environment and animals as well as for their hazardous health effects. As a result, new congeners of PFAS have rapidly replaced the so-called “old long-chain PFAS” (mainly PFOA and PFOS), currently out-of-law and banned by most countries. These compounds derive from the original structure of “old long-chain PFAS”, by cutting or making little conformational changes to their structure, thus obtaining new molecules with similar industrial applications. The new congeners were designed to obtain "safer" compounds. Indeed, old-long-chain PFAS were reported to exert thyroid disruptive effects in vitro, and in vivo in animals and humans. However, shreds of evidence accumulated so far indicate that the “restyling” of the old PFAS leads to the production of compounds, not only functionally similar to the previous ones but also potentially not free of adverse health effects and bioaccumulation. Studies aimed at characterizing the effects of new-PFAS congeners on thyroid function indicate that some of these new-PFAS congeners showed similar effects.

**Purpose:**

The present review is aimed at providing an overview of recent data regarding the effects of novel PFAS alternatives on thyroid function.

**Results and conclusions:**

An extensive review of current legislation and of the shreds of evidence obtained from in vitro and in vivo studies evaluating the effects of the exposure to novel PFOA and PFOS alternatives, as well as of PFAS mixture on thyroid function will be provided.

**Supplementary Information:**

The online version contains supplementary material available at 10.1007/s40618-024-02339-w.

## Introduction

Perfluoroalkyl and polyfluoroalkyl substances (PFAS) are a family of manufactured chemicals used worldwide. Their typical fluorocarbon structure confers their thermal resistance to high temperatures and repellency for water and oil, making them useful for the production of industrial and consumer products. The stability of their tight chemical bonds makes PFAS very resistant and difficult to eliminate, thus, persistency and bioaccumulation both in the environment and the human body, have led to the use of the term “*forever chemicals*” when referring to PFAS [11, 42, 53, 61]. Industrial manufacturing releases them into the environment, ending up in the food chain and accumulating in human bodies through the diet. Human dietary exposure to PFAS happens through vegetables and food of animal origin, as well as through drinking water consumption [41, 42]. The use of PFAS started in the last century, but concerns regarding their potential adverse effects on human health raised only in the last 20 years. The Environmental Protection Agency (EPA) has estimated over 9000 different PFAS, therefore, over the last decade, concerns have continued to mount and several studies demonstrating the harmful effects of these compounds on the environment and, in turn, on human health have been published [54]. Epidemiological and in vitro studies reported that some members of the PFAS family (in particular Perfluorooctanoic Acid PFOA and perfluorooctane sulfonic acid PFOS) were proven to: (i) increase blood cholesterol levels; (ii) decrease fertility in women; (iii) cause low infant birth weight; (iv) be potentially carcinogenic [110]. For these reasons, the so-called legacy- PFAS (mainly represented by the above-mentioned PFOA and PFOS) were phased out of production thanks to several legal restrictions [54]. Nonetheless, new PFAS were introduced by industries to substitute the banned ones [10]. Among the adverse effects on human health, PFAS are suspected to exert endocrine and in particular thyroid-disrupting effects. Thyroid diseases affect an estimated 200 million people worldwide and are also increasingly associated with a greater risk of developing other clinical conditions, including cardiovascular disease, metabolic syndrome, obesity, as well as depression, and anxiety disorders [23, 84, 124]. A recent review published in 2020, reported the thyroid-disrupting effects of both legacy and new-generation PFAS, showing mainly data regarding the banned PFOA and PFOS and few data regarding Hexafluoropropylene oxide-dimer acid (GenX) and 2-[(5-methoxy-1,3-dioxolan-4-yl)oxy], ammonium salt (C6O4), two PFOA novel alternatives [27]. At present, more and more studies focused on the potential thyroid adverse effects of the novel alternatives to PFOA but also novel alternatives to PFOS thus, providing the scientific community with more data regarding the safety, or toxicity for thyroid homeostasis of the new-generation PFAS. The present review aims to overview available data regarding the novel alternatives to PFOA and PFOS and their potential thyroid-disrupting effect. To exhaustively address the topic, we will initially provide a brief section on the more recent law restriction on PFAS use, following which, most recent studies regarding the thyroid-disrupting effects of these novel compounds and why PFAS exposure is detrimental to human health, will be overviewed.

## Regulations and laws issued in recent years

Other than the out-of-law of PFOA and the inclusion of PFOS in the list of potential carcinogens, numerous restrictions have emanated in the last decade. In 2019, the Stockholm Convention included PFOA, its salts, and PFOA-related compounds as substances to be eliminated. POPs Regulation prohibits the use of PFOA, since July 2020 [118]. In addition, in 2020, Annex I entry for PFOS was amended to remove exemptions no longer needed in the European Union (EU) [19]. In December 2023, the international agency for research on cancer (IARC) considered PFOA to be carcinogenic to humans and included it in Group 1 (Cancer).

Numerous PFAS are regarded as substances of an equivalent level of concern (ELoC) to carcinogens, mutagens, toxic and repro-toxicants, persistent/very-persistent, bio-accumulative/very-bioaccumulative. Thus, were included in the “Registration, Evaluation, Authorisation and Restriction of Chemicals” (REACH) candidate List of substances so-called “of very high concern” (SVHC) [98]. Indeed persistence, mobility, and toxicity of these contaminants represent a wildlife warning and also to human health when exposed to the environment (including through drinking water). There are overarching objectives in EU water legislation, including the protection of human health and the environment from the combined effects of toxic and persistent pollutants. The Water Framework Directive identified the main substances that pose a risk to the aquatic environment and established environmental quality standards for them [18]. In December 2019 the European Parliament and the Council agreed on the recast of the Drinking Water Directive, by establishing a limit of 0.5 µg/l for all PFAS [97]. In October 2020, the European Commission published the Chemicals Strategy for Sustainability which includes phasing out the use of PFAS in the EU [17]. In September 2020, the European Food Safety Authority (EFSA) concluded that the main PFAS that accumulate in the body are PFOA, PFOS, perfluorononanoic acid (PFNA), and perfluorobutane sulfonic acid (PFHxS). In this view, for the Ʃ of these for PFAS, a total tolerable weekly intake (TWI) was limited to 4.4 ng/kg of body weight per week [50]. However, the exposure of a relevant proportion of the European population to those substances exceeds the TWI, thus, raising concern. The Netherlands, Germany, Norway, Denmark, and Sweden, submitted the restriction proposal to ECHA on 13 January 2023 to cover a wide range of PFAS uses [48]. In 2021, Canada proposed to consider long-chain perfluoro carboxylic acids (C9–C21 PFCAs) for inclusion in the Stockholm Convention, moreover, since 2022, PFHxS, its salts, and PFHxS-related compounds have been included [47].In surface waters, PFOS is already listed as a priority substance, but other PFAS are now also recognized to pose a risk. In most recent regulations, the term PFAS refers to a “summation of 24 PFAS”, which was calculated by comparing the potency of the substances with that of PFOA (Supplementary Table 1). The proposed Environmental Quality Standard for the Ʃ of PFOA equivalents is expressed as an annual average (AA) value of 0.0044 μg/L in surface waters [117]. On 12 January 2021 took effect the recast of the Drinking Water Directive, which included limits for PFAS providing two parametric values: “Ʃ of PFAS” of 0.1 μg/L for 20 PFAS and “PFAS Total” of 0.5 μg/L [119]. In the United Kingdom UK, there is no specific standard listed in the Water Quality Regulations for PFAS but the Drinking Water Inspectorate requires water companies to monitor a list of 47 PFAS for risk assessment purposes and take appropriate action when PFAS detected. Moreover, The Health and Safety Executive recently recommended that the government should develop statutory guidance for PFAS in drinking water [76]. Commission Regulation (EU) 2022/2388, amending Regulation (EC) No 1881/2006, sets individual maximum levels for PFOS, PFOA, PFNA, and PFHxS, together with a maximum level for the sum of those PFAS, in foods of animal origin [20]. Member States must test the levels of PFAS in domestically produced and imported food and foods with levels of PFAS higher than those allowed cannot be sold in the EU. Commission Recommendation (EU)2022/1431, in force from September 2022, recommends that Member States should test for the presence of the same 4 PFAS during the years 2022, 2023, 2024, and 2025 in a wider range of foodstuffs as compared to those covered in the recommendation 2022/2388 [21]. Commission Implementing Regulation (EU) 2022/1428, in force from September 2022, provides methods of sampling and analysis for the control of PFAS in certain foodstuffs and provides acceptance criteria for validation of methods and information on reporting and interpretation of results [22].

Environment Protection Agency (EPA) stated, on March 2023, the proposed National Primary Drinking Water Regulation (NPDWR) for PFOA, PFNA, hexafluoropropylene oxide dimer acid (HFPO-DA, commonly known as GenX Chemicals), PFHxS, PFOS, and perfluorobutane sulfonic acid (PFBS). By the end of 2023, the EPA will expect to finalize this regulation, in addition, it will be expected a prevent thousands of deaths and reduce PFAS-attributable illnesses thanks to the implementation of this rule [55].

Information reported indicates that a continuous evolution of international restrictions regarding PFAS is ongoing. However, with regard to novel alternatives of PFOA and PFOS, few data are available, and current restrictions were adopted only for GenX, as herein reported. For these reasons, studies on alternatives to restricted PFAS are strongly encouraged.

## How can PFAS interfere with thyroid function?

Thyroid hormones (THs) are important regulators of the growth, development, and metabolism of all vertebrates [59]. The hypothalamus produces the “thyrotropin-releasing hormone” (TRH), which stimulates another gland, the pituitary, to release thyrotropin (thyroid-stimulating hormone, TSH). TSH is released in the bloodstream circulation and in turn stimulates the thyroid gland to produce thyroid hormones (THs) [101]. Thyroxine (T4) and triiodothyronine (T3) are the main THs produced by the thyroid gland. THs biosynthesis and release, as well as several proteins expression and activities, are stimulated by TSH (the solute carrier family 5A, also known as sodium-iodide symporter (NIS), pendrin (PDS), dual oxidase type 2 (DUOX), thyroid peroxidase (TPO), thyroglobulin (Tg) and deiodinases type 1 (D1, DIO1), 2 (D2, DIO2) and 3 (D3, DIO3)). In physiologic balance, T4 and T3 regulate their concentrations in the blood by negative feedback acting at the hypothalamic and pituitary levels [102]. THs are transported via various blood proteins (e.g., albumin and mainly thyroxine-binding globulin (TBG) or primarily transthyretin (TTR) to peripheral tissues). The primary action of THs in cells is to control gene transcription through the activation of nuclear receptors [102].

**Fig. 1:**
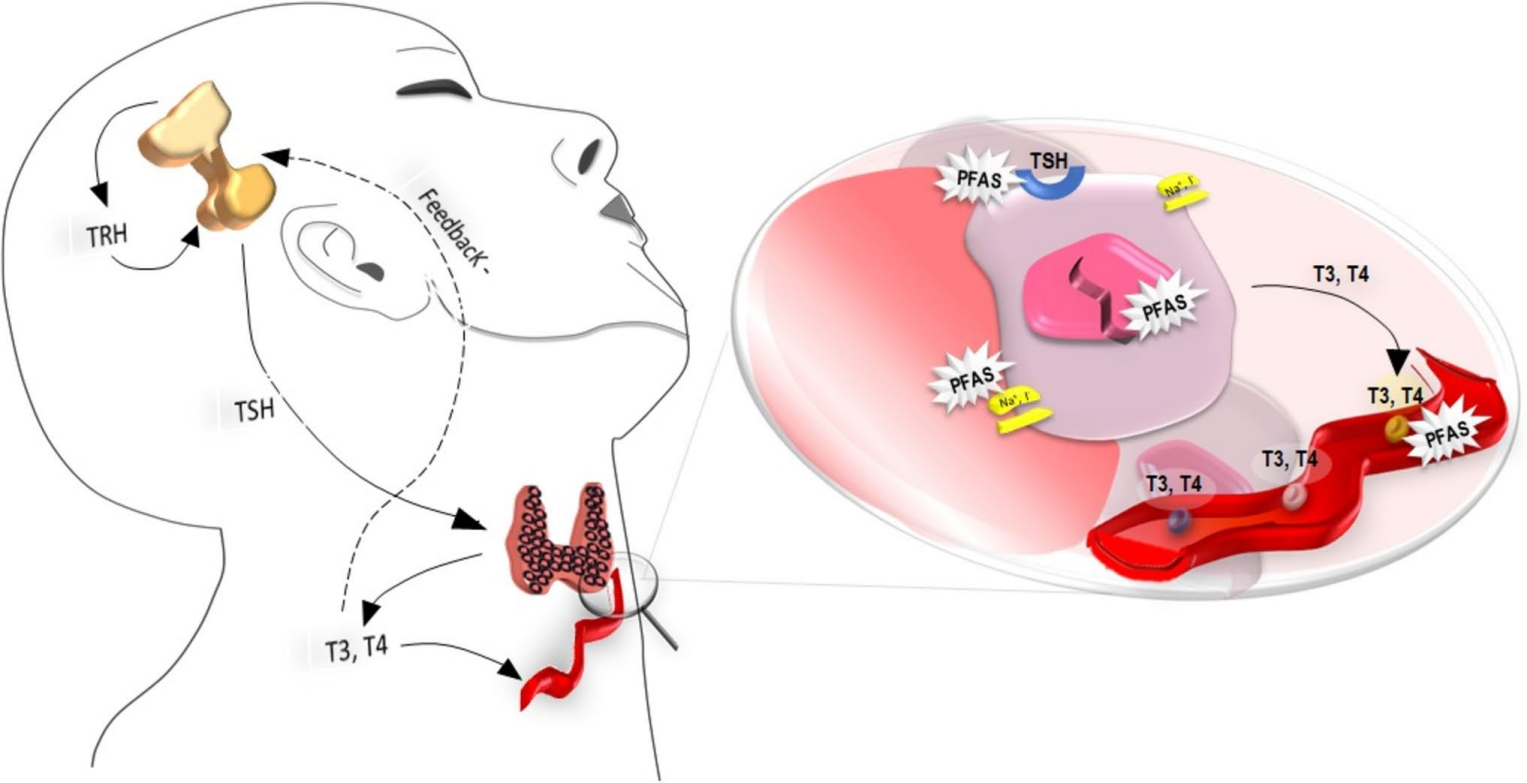
Interference of PFAS with thyroid function. The image shows the hypothalamic–pituitary–thyroid axis with the negative feedback exerted by thyroid hormones on TSH secretion. The synthesis and secretion of thyroid hormones can be influenced by PFASs through various mechanisms: (i) inhibition of iodine uptake via interaction with NIS, (ii) alteration of Tg synthesis, (iii) alteration of TPO, (iv) alteration of the signalling pathway of thyroid hormones

In 2018, an update in the Organization for Economic Co-operation and Development test guidelines (OECD TG 408) was made. These test guidelines are related to exposure tests on animal. The up-date included the measurement of TSH, T4, and T3 serum levels, as well as thyroid gland volume. This choice was made to improve the detection of potential endocrine toxicity since these newly included parameters are responsive to the perturbation of the thyroid axis [96]. The basic areas of interference with thyroid metabolism are (i) inhibition of the uptake of iodide at the cellular membrane of thyrocytes by blocking the NIS transporter; (ii) synthesis inhibition via TPO; (iii) binding of transport protein TTR in the bloodstream; (iv) altered hepatic phase-2 catabolism by glucuronosyltransferase and sulfotransferase metabolism of T3 and T4; (v) alteration of deiodinase-regulated T4 metabolism; (vi) alterations of transport across cellular membranes and alteration of cellular receptors (TSH receptor); (vii) epigenetic regulation, such as DNA methylation changes [51, 68, 72, 103]. In vitro and in vivo data demonstrated that different PFAS could interfere at several of these levels causing thyroid disruption [31, 64, 77, 100, 116]. In particular, it is important to highlight that recent studies demonstrated that the specific structure of PFAS, which is essential for giving PFAS its unique chemical characteristics, would play a crucial role in thyroid disruption. Indeed, functional groups and carbon-chain length of PFAS have been found to influence their binding affinities to THs or receptors. In particular, PFASs can compete with T4 for binding to the human TTR which may reduce THs levels leading to endocrine-disrupting adverse effects. A recent study, using a computational approach, showed that the structural determinants of PFASs are the carbon-chain length and functional group, indeed longer carbon-chains of PFASs and sulfur-containing tend to interact in a stronger manner than the shorter-chains. Interestingly, a high binding ability was reported also regarding “short-chain” PFAS, moreover, their interaction energy is similar to the longer-chain PFAS. Taken together it could be hypothesized that PFASs composed of a short chain are not totally safe, thus they should be carefully regulated [38]. Another thyroid target of PFAS is suspected to be the NIS. The recent study by Stoker et al., tested the ability of 149 unique per- and polyfluoroalkyl substances (PFAS) to inhibit the NIS activity, that is the transport of iodine. Among this set, 38 out of 149 (25.5%) of the PFAS chemicals inhibited the uptake of iodide ≥ 20% as showed by the multi-concentration testing, with 25 out of these 38 displaying a ≥ 50% inhibition, being PFOS and PFHxS recognized as the most powerful inhibitors, even if the significant reduction was observed also for several other screened PFAS chemicals [111]. The binding of T3 to TR regulates TRα and TRβ gene expression [128]. Evidence suggests that PFASs exhibited agonistic activity on the TR pathway PFASs may not exploit only one mechanism, but one suggested cause is that they directly bind to TRs and promote target gene expression. This explanation sheds light on the possible molecular mechanisms of PFAS toxicity [134]. The various targets of PFAS in the thyroid gland are shown in Fig. 1.

## Is PFAS exposure an environmental risk factor for the development of thyroid cancer?

Recently PFOA entered the list of carcinogens for humans (Cancer; [120]). However, the issue of whether exposure to PFAS could specifically increase the incidence of thyroid cancer remains somehow controversial. Indeed, even if in vitro studies suggested a potential relationship between PFAS exposure and thyroid cancer development, data from epidemiological studies showed controversial findings. Early studies, aimed at investigating the association between PFAS exposure and the incidence of several cancers, but not specifically thyroid cancers, reported a relative low risk for thyroid cancer development in exposed workers. However, it should be considered that: (i) a very limited number of subjects were enrolled; (ii) the findings should not be regarded as consistent in that a great discrepancy was present between studies [6, 26, 109, 121]. On the other hand, also the results provided by more recent large series studies, which, at least in some cases, were specifically designed for assessing thyroid cancer risk following PFAS exposure, have not solved the controversy. Indeed, some studies suggest a potential correlation between circulating PFAS levels and increased thyroid cancer risk, while some others found a negative association or no association.

Just to give a few examples, van Gerwen et al., investigated the potential association between PFAS exposure and thyroid cancer. The levels of PFAS were evaluated in plasma samples of patients collected before or at thyroid cancer diagnosis. They found that exposure to several PFAS and in particular to n-PFOS may be associated with an increased risk of thyroid cancer [120]. Messmer et al., showed that, in a population exposed to PFOA (Merrimack, Massachusetts, drinking water contaminated with PFAS), the risk of thyroid cancer (and of other three type of cancers) was significantly higher compared to controls living in low-exposure areas [85, 93]. Moreover, in an ecological study, Alsen et al. [4], showed, that high serum levels of PFNA and PFOA (among other PFAS including PFOS, PFHxS, PFHpA or PFBS) were the only to be associated with increased thyroid cancer incidence in the US.

On the other hand, Haoran et al., found a significant negative associations between high serum levels of PFOA, PFNA, PFHxS, PFDA, and PFUnDA and thyroid cancer risk. One recent cross sectional study was conducted to evaluate associations between exposure to various chemicals and previous endocrine cancer diagnoses. In this study (2015–2018), seven PFAS (PFHS), (MPAH), (PFDE), (PFNA), (PFUA), (PFOA) and (PFOS) were measured in the serum of participants (non-institutionalized, nationally representative, sample of children and adults used to assess the health and nutritional status of the United States population, NHANES). The results demonstrated an inverse association between PFOS exposure and the likelihood of a prior diagnosis of thyroid cancer among women, whereas all other associations with thyroid cancer were null [16].

By moving onto in vitro data, several studies suggested a possible relationship between PFAS exposure and thyroid cancer. Just to give few examples it was demonstrated that PFOA, PFHxA, GenX and ADONA could exert pro-tumorigenic effects on thyroid cancer cells in vitro by enhancing some tumor-related genes, affecting cell proliferation, inducing genotoxicity or modulating the secretion of pro-tumorigenic chemokines [28, 30, 132]. Thus, whereas in vitro data seem to indicate a potential pro-tumorigenic effects, at least for some PFAS on thyroid cancer, epidemiological studies still require more data to reach a conclusive answer.

### New PFOA and PFOS alternatives: restyling of legacy-PFAS

The extraordinary versatility of PFAS and their practically indestructible chemical structure, as well as their low production cost, has prompted industries to rush to immediately replace the restricted ones like PFOA and PFOS with alternatives, the so-called “new alternatives to PFOA and PFOS” or new/next generation PFAS. Compared with their banned predecessors (PFOA and PFOS), there is substantially less information regarding emerging PFAS in the environment. It is important to point out that the term "emerging PFAS" could be not theoretically correct for identifying the available alternatives to PFOA and PFOS, currently used by industries. Indeed, among these alternatives, there are not only new compounds but also: (i) old molecules that were less used previously; (ii) long-chain PFAS less studied and not subjected to restrictions, and (iii) legacy PFAS “masked” by new PFAS by either shortening or changing the linearity of their original structure. Indeed, the “classic” perfluorinated compounds, such as PFOS/PFOA, consist of a fully fluorinated alkyl chain with a functional group (e.g. sulphonate) at the end. The new PFAS instead can have either a short-chain (perfluorocarboxylic acid with carbon number < 8 and perfluorosulfonic acid with carbon number < 6) or a slightly modified structure, for example, being ether or cyclic molecules, which could not meet the definition of new molecules [53, 56] (Supplementary Table 1). These compounds emerged to meet market demand and are increasingly found in environmental and human matrices at relatively high concentrations due to their up-to-now unrestricted use [11]. The environmental presence of these alternatives is the result of direct emissions from fluorochemical manufacturing PFAS. Unfortunately, the available studies regarding these compounds showed in most cases comparable or even greater toxicity in both epidemiological and toxicological studies [39, 52, 105].

In vitro and in vivo evidence showed that legacy PFAS and some novel PFOA and PFOS alternatives could be potential thyroid interferents [27]. These novel alternatives are numerous and some of them are still unknown to the scientific community since no studies are available on them. For this reason, the present review will take into account the currently most studied PFOA and PFOS alternatives and their effects on thyroid homeostasis.

Table 1 shows the most studied alternatives to PFOA and PFOS available and a description of each compound as well as its known effects on the thyroid which will be overviewed in the following paragraphs.

**Table 1:**
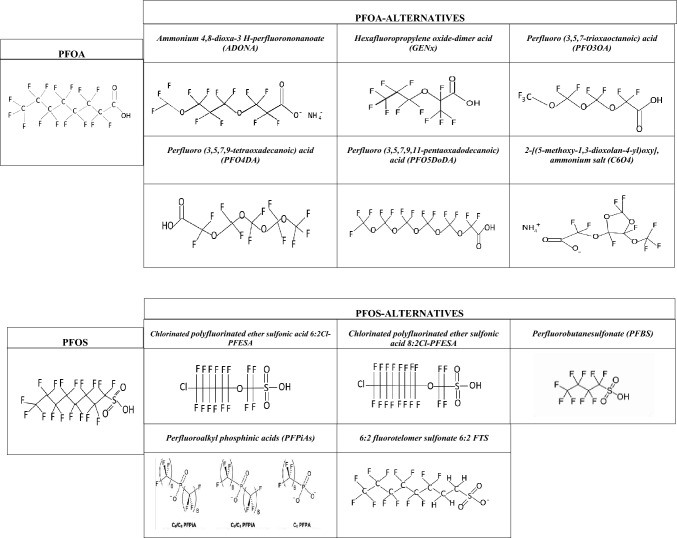
The most studied alternatives to PFOA and PFOS showed with respective chemichal structures and names

## Effect of novel PFOA alternatives on the thyroid gland

### Perfluoroalkyl ether carboxylic acids (PFECAs)

PFECAs are a PFAS subclass that has become an increasing environmental focus. They are composed of active carboxyl groups and contain a perfluoropolyether chain that confers hydrophobic properties [40, 112].

Similar to PFOA alternatives, PFECAs have become widely used in fluoropolymer manufacture or are produced as by-products during this process. Since their introduction, many of these alternative chemicals have been detected at relatively high levels in surface water and human blood [123].

### Ammonium 4,8-dioxa-3 H-perfluorononanoate (ADONA)

ADONA use is applied in the production of some fluoropolymers and fluoroelastomers and serves as an emulsifier. The final result is a compound functionally indistinguishable from the previous ones made by the use of the legacy PFOA. ADONA is an ammonium salt of perfluoro-4,8-dioxo-3H-nonaoic acid and is constituted by the 7-carbon perfluoropolyether chain and the ammonium ion [60, 67]. ADONA is employed in fluoropolymer manufacturing and is mainly recovered from waste streams or destroyed thermally during the manufacturing process. ADONA is used as an emulsifier in some fluoropolymers, such as polytetrafluoroethylene (PTFE), widely used as a non-stick coating on cookware and other surfaces that come into contact with food. Few data are available in humans on serum concentrations of ADONA. In one study performed in plasma samples of blood donors living in South Germany, the compound was generally detected at levels above the Limit of Quantitation (LOQ) of (0.2–14.4 μg/l) only in a minority of samples [60]. Little information is available for ADONA levels in drinking water. When PFOA was phased out, in 2008, the waters of river Alz showed 6.2 μg/l of ADONA, compared to 7.5 μg/l of PFOA detected in 2006 at the same sampling site. Of note, in 2009, the ADONA concentrations in the same river declined to 0.8 μg/l [1].

Wistar rat repeated dose toxicity test showed increased incidence and severity of thyroid follicular hypertrophy up to 100 mg/kg in males; the authors considered this an adaptive change to the increases in liver weight and incidence and severity of hepatocellular hypertrophy/hyperplasia [45].

In an in vitro study performed on both human normal thyroid cells (NHT) and rat thyroid cells (FRTL5), ADONA showed minimal adverse effects on cell viability and proliferation [132]. However, it was demonstrated that the expression of THs regulation genes was altered in vitro by ADONA. Indeed*,* it was found that ADONA altered the mRNA levels of PAX8 in FRTL5 and of TSHr in NHT at a sub-cytotoxic dose [132]. Pax8 belongs to the paired-box (PAX) family. This transcription factor is involved in the development, and differentiation of the cells composing the thyroid gland, and hormone synthesis [33, 34]. In addition, PAX8 is reported to be up-regulated in thyroid cancer being regarded as a gene potentially involved in thyroid carcinogenesis [43]. TSH-receptor (TSH-R) plays a crucial role in the synthesis of THs and also in their release [78]. Damages or incorrect expression of this gene could lead to several thyroid-related diseases (cancer, autoimmune diseases, and many others) [78]. The mechanism by which ADONA could interfere and disrupt thyroid function is still not yet elucidated but its adverse effect was demonstrated in vitro [132].

### Hexafluoropropylene oxide-dimer acid (GENx)

Gen-X (also known as HFPO-DA) is used, following the gradual elimination of PFOA [9]. Gen-X is composed of oxide-dimer acid with 6 carbon atoms linked to eleven fluorine atoms. This compound was estimated to have a 10–100 tons annual production in Europe, where it has been in use for more than ten years [44, 104, 114]. High levels of GenX were found in river sampling sites from Germany and The Netherlands (EU) both nearby and far away from fluoro-chemical plants. In addition, high levels were detected in drinking water and raw water of plants intended for drinking water treatment [9, 62, 74]. In 1980 the Fayetteville Works facility (North Carolina) contaminated the Cape Fear River by discharged process wastewater [79, 122]. To provide an exhaustive answer to the question: “Are PFAS detectable in me?” it was developed the so-called “GenX Exposure Study”. In response, New York City State and researchers collected blood samples (289 adults and 55 children among Wilmington residents) between 2017 and 2018. Additionally, 44 subjects had a second blood sample collected six months after the first one. The first evaluation was aimed at detecting which PFAS were present. The second one was performed to highlight changes in PFAS levels over six months. It should be highlighted that GenX was not detectable despite its presence in drinking water in the same area [79].

The thyroid adverse effects exerted by GenX are demonstrated both by in vitro studies and animal models. Briefly, GenX had a greater incidence of placental abnormalities on mice while in rats it was observed a greater incidence of reduced maternal THs levels as well as elevated levels of Peroxisome proliferator-activated receptors (PPAR)-regulated the expression of both maternal and fetal genes expressed in livers. Moreover, GenX was demonstrated to be a developmental toxicant for rats causing increased neonatal mortality and reduced birth weight [25].

In the recent study by Zhang et al., the potential adverse effects of ADONA GenX and PFOA in vitro on human and rat thyroid cells were compared [132]. The results identified GenX as the compound showing the highest thyroid cytotoxicity. In addition, GenX exposure was associated with a more profound reduction of the proliferation rate of cells showing an even more profound adverse effect when compared to PFOA. In addition, Genx was able to induce PAX8 and TSHR mRNA expression levels in both FRTL-5 and normal thyroid cells [132]. The data by Zhang et al., confirmed a previous study by Coperchini et al., showing strong genotoxicity and cytotoxicity of GenX for rat thyroid cells in vitro [28]. Taken together the above data from in vitro studies and in animal models point toward an adverse effect of GenX on thyroid homeostasis.

### Other PFECAs: perfluoro (3,5,7-trioxaoctanoic) acid (PFO3OA), perfluoro (3,5,7,9-tetraoxadecanoic) acid (PFO4DA), and perfluoro (3,5,7,9,11-pentaoxadodecanoic) acid (PFO5DoDA)

The perfluoro(3,5,7-trioxaoctanoic) acid (PFO3OA), perfluoro(3,5,7,9-tetraoxadecanoic) acid (PFO4DA), and perfluoro(3,5,7,9,11-pentaoxadodecanoic) acid (PFO5DoDA) were investigated for their potential thyroid disruptive effects. Median blood concentrations of PFO4DAand PFO5DoDA from healthy volunteers living near the Chemours plant (North Carolina, USA) have been detected at > 2 ng/mL (detection rate:98%) and < 0.5 ng/mL (detection rate: 98%), respectively [12]. PFO2HxA, PFO3OA, and PFO4DA have been detected in surface water from the Cape Fear River (PFO4DA: 2.5 ng/ml) [79]. In a recent study, the developmental toxicities of various PFECAs in zebrafish embryos (e.g., perfluoro(3,5,7-trioxaoctanoic) acid (PFO3OA), perfluoro(3,5,7,9-tetraoxadecanoic) acid (PFO4DA), and perfluoro(3,5,7,9,11-pentaoxadodecanoic) acid (PFO5DoDA) was compared with that of the banned PFOA. Results showed that based on half maximal effective concentrations (EC50), an increase in the toxicity was observed showing a higher effect of PFO5DoDA when compared to the others and also with PFOA. However, a toxic effect was observed also after exposure to PFO4DA, PFOA, or PFO3OA, showing uninflated posterior swim bladders that were the most frequently observed malformation [123]. PFECA exposure lowered in a significant manner THs levels (e.g., T3 and T4) in the whole body of larvae at 5-d post-fertilization following disrupted the metabolism of TH. This effect was similar to what was observed after exposure of larvae to PFOA. In addition, the transcription of UDP glucuronosyltransferase 1 family a, b (ugt1ab), a gene related to the metabolism of THs, increased in a dose-dependent manner. Finally, the supplementation of exogenous T3 or T4 partly rescued the posterior swim bladder malformation induced by PFECAs. This study reported for the first time a thyroid-disrupting effect exerted by emerging PFECAs as causing swim bladder malformation. Thus, adverse effects were caused also by these PFOA alternatives for the thyroid system [123].

### The PFAS with a cyclic structure: 2-[(5-methoxy-1,3-dioxolan-4-yl)oxy], ammonium salt (C6O4)

C6O4 is a relatively new compound, for which several studies have been performed in the last three years. Indeed, before 2020 no peer-reviewed study was available regarding the potential adverse effects of this compound for human health. C6O4 use applies to the polymerization process of fluoropolymers for the final make of food contact articles [49]. C6O4 is not thermally stable and shows a high degree of solubility. C6O4 was shown to be produced or imported in a quantity near 1–10 tons per year in the EU [46]. In 2019 C6O4 was found in the River Po by ARPAV (the Regional Agency for the Prevention and Protection of the Environment in Veneto, Italy) and was also reported to be found with peak concentrations of ~ 100 ng/l near some areas of the Veneto region (Italy), [5]. No available data on serum or tissue levels of the new generation perfluoro-alkyl substance C6O4 exist so far. However, C6O4 has been measured in the serum of MITENI workers, with very variable concentrations and very high maximum concentrations, being the highest median value (60.77 ng/mL) found in workers of the pilot plant for C6O4 recovery [65]. As far as the thyroid axis is concerned, the first in Vitro study on C6O4 effects on thyroid cells was performed in 2020 by Coperchini et al., who evaluated the effects of C6O4 exposure in FRTL5 rat thyroid cell line, showing no relevant adverse effects at difference with what observed after exposure to the long chain PFOA and PFOS [29]. However, in a more recent study by the same group, it was found that some PFAS, including C6O4, exert an up-regulation of the pro-tumorigenic chemokine CXCL8 at mRNA and/or protein levels [30]. Furthermore, another in vitro study evaluated the potential disrupting effect of C6O4 (as compared with PFOA and PFOS) on a murine thyroid cell model, showing that C6O4, PFOA, and PFOS could exert a potential direct differential interaction on the same binding site of the extracellular domain of TSHR. On the other hand, no effect on either cell viability or on cAMP levels and iodine uptake was exerted by C6O4 in this in vitro model [36].

## Effect of novel PFOS alternatives on the thyroid gland

### Chlorinated polyfluorinated ether sulfonic acid (PFESAs), the components of F-53B

Chlorinated polyfluorinated ether sulfonic acid 6:2Cl-PFESA and 8:2Cl-PFESA are two major components of the commercial compound F-53. Based on the metal-plating industry.6:2 Cl-PFESA has a perfluoroalkyl ether chain with 8 carbon atoms, the sulfonic acid group in position 2, and an atom of chlorine in position 6 of the chain. 8:2Cl-PFESA is constituted by a perfluoroalkyl ether chain with 10 carbon atoms, a sulfonic acid group in position 2, and an atom of chlorine in position 8.F-53Bhas been widely used in decorative and hard metal plating since the late 1970sand was applied as a mist suppressant in the electroplating industry [126]. Following global actions to phase out PFOS, F-53B has represented a good market valuation in China owing to lower production costs [107]. Although China is the only known country producing Cl-PFESAs, they were detectable in Arctic wildlife, indicating the potential for long-distance transport and possibly worldwide contamination of Cl-PFESAs [63], which was shown to bioaccumulate in aquatic wildlife at concentrations comparable to PFOS [37, 125].

Next to PFOS, 6:2 Cl-PFESA has become the third most prevalent PFAS detected in sera blood or cord blood of pregnant women in China [88]. As far as the potential thyroid-disruption effect of PFESAs is concerned, some studies reported interesting results on animal models. Indeed, one of the main concerns regarding the disruptive effects of F-53B on the endocrine system is the dysfunction of the hypothalamic–pituitary–thyroid axis.

A toxicity study, performed after 28-d continuous oral administration of F-53B to rats [73], demonstrated a reduction of T3 and T4 in the absence of TSH changes, in line with previous studies on oral administration of other PFAS in rat models, reporting a decrease in thyroid hormone levels without compensatory increases in TSH [94, 95]. Thyroid histology of rats treated with F-53B confirmed the presence of thyroid follicular hyperplasia, as well as increased protein expression of TSHR and TPO by immunohistochemistry [73].

On the contrary, when the zebrafish model was used, different findings were observed. Deng et al. exposed zebrafish embryos to different concentrations of F- 53B for 5 days finding increased levels of T4 [37]. Similar findings were reported in the study by Shi et al. that observed, an increase in T4 levels in both in adult and embryo zebrafish, following exposure to F-53B for 180 days. The same study also found a decrease in T3 levels in adult fish and larvae after F-53B exposure [108]. However, moving to other aquatic species, different trends were observed. Indeed, T3 and free T3 levels were increased in Chinese rare minnows after 28-d exposure to F-53B [91]. Moreover, mRNA expression of genes regulating thyroid hormone synthesis including TSH, NIS, Tg, TPO, TTR, deiodinase (Dio1, Dio2), receptor (TRα and TRβ), and CRH were decreased. In addition, the uridine diphosphate glucoronosyl-transferases (UGT1A) gene, regulating thyroid hormone metabolism, was also downregulated. After twelve weeks of depuration without exposure, the perturbation of these genes persisted, demonstrating that chronic developmental exposure to Cl-PFESAs caused persistent TH-disrupting effects in fishes [91].

These data would suggest that the abnormal transcription of thyroid hormone receptor genes, with, however, some species-related differences, could be ascribed as the mechanism involved in the endocrine toxicity exerted by F-53B.

Moving to in vitro data, the exposure of human HEK 293 embryonal cells to F-53B showed a perturbation of TH levels. The reason is that F-53B could not only fit into the binding pocket of TRα and TRβ, like T3 but could also fit into the TTR ligand-binding pocket, similar to T4[130]. Indeed, it was demonstrated that in vitro, F-53B exhibits a higher affinity to transthyretin (TTR) than PFOS and can activate TRs [129]. Finally, moving to in vivo data on humans, the potential association between Cl-PFESA serum levels and thyroid cancer was investigated in a case–control study revealing no significant association. However, a significant association was found in the same cohort between Cl-PFESA levels and increased FT4 serum concentrations [90].

In a study population performed on subjects from “Isomers on C8 health project in China”[133], it was reported that exposure to Cl-PFESAs, which was detected in 64.6–99.9% of the population, was positively associated with thyroid hormones concentrations in the general adult population, being this association non-linear. Taken together these two Cl-PFESA, which compose F53B, are strongly suggested to exert thyroid interference in vitro, in several animal models, and also in vivo on humans.

### Perfluoroalkyl phosphinic acids (PFPiAs)

The chemical structure of PFPiAs is very like to the one of the legacy PFOS [83]. They are usually used as wetting and leveling agents in cleaning products and were historically used as pesticides in the United States until 2008 with a consistent annual production volume (i.e. 4.5–227 tons between 1998 and 2002) [75]. PFPiAs have been found in different matrices including human bodies, waters and also in dust, and others, [35, 70, 77], and 6:6, 6:8, and 8:8 PFPiA were the most abundantly detected homologs. The 6:6 and 6:8 PFPiA were detected in over 50% of 50 human sera samples (range of 4–38 ng/L) [82]. The mean concentrations of the summation of these PFPiAs (6:6, 6:8, and 8:8 PFPiA) in samples of waste recovered in Vancouver was 2.3 ng/g, and 1.87 ± 2.17 ng/g wet weight in fish, dolphin, and bird plasma in North America [35]. Several studies demonstrated that PFAS with longer carbon chain lengths (like PFPiAs) are more toxic than short-chain PFAS [80, 81]. In the aquatic animal model, it was reported that PFPiAs (6:6, 6:8, and 8:8) modulate the expression of some genes in the HPT axis and cause THs imbalance, demonstrating their effect on thyroid disruptors. Indeed, after exposing a zebrafish to the 8:8 PFPiA it were showed not only higher levels of T3 and T4 but also other adverse effects which could be due to thyroid interference (lower body length, and heart rate). Interestingly these three PFPiAs inhibited the expression of tsh and tshβ genes by a negative feedback mechanism, showing in particular a higher effect exerted by 8:8 PFPiA [89]. In a subsequent study by Kim et al., zebrafish embryos (b4 hpf) exposed to various concentrations of 8:8 PFPiA for 144 days, showed an up-regulation of several genes involved in neurodevelopment (crhb, dio3a,tshr, and nkx2.1)[77]. Furthermore, global DNA methylation was significantly decreased. The promoter of DNA methylation of selected genes (dio3, tshr, nkx2.1) was not statistically modified, but dio3 methylation showed a decreasing trend after 8:8 PFPiA exposure. Taken together, data on PFPiA are available only on aquatic animal models and suggest a potential thyroid disruption by these PFAS. However, to make a firm point on these novel alternatives more studies are required.

### Perfluorobutanesulfonate (PFBS)

PFBS is a short-chain substitute for PFOS. PFBS is composed of a 4-carbon perfluoroalkyl chain and a sulfonic acid functional group. PFBS is used in industrial products, such as textiles, firefighting foams, electronics, and plastics [115]. In vitro data on a thyroid cell model reported no changes in cell viability, proliferation, or cAMP levels after exposure to PFBS. On the other hand, when animal models are taken into account, PFBS causes multiple effects on the thyroid system. Feng et al. evaluated the interference of prenatal PFBS exposure on perinatal growth and development, pubertal onset, and reproductive and thyroid system in female mice orally administered on days 1–20 of gestation. They showed a decrease in serum T4 and T3 levels in fetal, pubertal, and adult offspring with a parallel even if moderate increase in the concentrations of TSH and TRH. Moreover, in PFBS dams, it was observed a total T4 and T3 levels and free T4 levels significantly decreased as well as an increase in TSH levels. These results indicate that prenatal PFBS exposure causes hypothyroxinemia [57]. In a more recent study, Cao et al. found that exposure to a quantity ≥ 200 mg/kg/day of PFBS through reducing THs-induced inhibition of Akt-mTOR-signaling in granular cells and cumulus cells of adult mice, led to impairment of the development of follicles and the biosynthesis of ovarian hormones [14].

PFBS is frequently found in the aquatic environment representing an increasing concern for the *habitat.* The multigenerational thyroid-disrupting potential of PFBS was investigated by examining three generations of medaka eggs (i.e. F0, F1, F2). PFBS exposure decreased the levels of T3 in F0 female blood. On the other hand, PFS was found to increase T3 or T4 levels in brains of F0, in that hyperthyroidism suppressed the local transcription of Dio2. In addition, the decrease of T3 was transferred to F1 eggs, nevertheless, a reversion of the parental influences was present in F1 larvae. High T3 levels in F1 larvae were coupled with delayed hatching [14]. Moving to, F1 adults they showed symptoms of thyroidal disruption that were comparable with F0 adults. Finally as regards F2, although an observation of a permanent thyroid disruption and synthesis of T4 in F2 larvae, it was found a low recovery. In conclusion, offspring, albeit not directly exposed, suffered from a more severe thyroid dysfunction [14]. In addition, a recent study in the same aquatic animal model showed that PFBS exposure caused interference with several processes of thyroid function (e.g., thyroid gland development, synthesis and transport of THs receptor-mediated signaling, and feedback regulation). The combinations of PFBS and hypoxia interacted to interfere with the correct function of the thyroid endocrine system [115]. Another study evaluated the effect of PFBS on zebrafish exposed to increasing concentrations of the PFAS for 4-d, with a concomitant addition of probiotic bacteria Lactobacillus rhamnosus in the rearing water. The results showed that probiotics inhibited the stunted growth defect of PFBS via stress and modulation of the thyroid axis [113].

Moving to human data, a recent prenatal perspective cohort study by Cao et al., found a negative association of cord serum PFBS with TSH concentration in newborns [15]. Taken together, data available for PFBS encompass in vitro, animal models, and in vivo data, which consistently point toward a thyroid disruptive effect of this PFAS.

### 6:2 Fluorotelomersulfonate: 6:2 FTS

One potential PFAS replacement based on reduced persistence and/or toxicity is 6:2 fluorotelomer sulfonate (6:2 FTS), which has been found in water at known PFAS-contaminated sites [8, 13, 24]. It is composed of a perfluoroalkyl chain with 8 carbon atoms and presents a telomeric group in position 6 and a sulfonate group.

Capstone FS-17, used in several commercial products with different applications (spacing from cleaning products to paints) is mainly composed of fluorotelomer sulfonates (FTS). Other applications include mist suppressant products in chrome plating (e.g., Fumetrol 21), and are currently used as replacements of PFOS and PFOA. 6:2 FTS is both an abiotic breakdown product of 6:2 fluorotelomer sulfonamide alkyl betaine (FTAB) and 6:2 fluorotelomer sulfonamide alkylamine (FTAA) and (D’Agostino and Mabury 2017) and a biotransformation product of 6:2 fluorotelomermercaptoalkylamido sulfonate [71]. Biomonitoring California group for chemicals analyzed the 6:2 FTS levels in the serum of 100 Chinese and Vietnamese adults living in the San Francisco Bay area collected in 2016 and 2017 reporting no significant concentrations of this compound [13].

In vitro data suggest that 6:2 FTS may be involved in altering the binding of THs to serum carrier proteins, however, 6:2 FTS was estimated to be 100 times less potent than PFOS as a thyroid hormone disruptor [7]. In a recent study, Flynn et al. evaluated if PFAS exposure (including 6:2 FTS) could affect thyroid morphology and function in toads and salamanders finding no significant alterations of thyroid gland histology and/or in thyroid hormone levels. However, the low number of samples could be a limitation of this study as suggested by the Authors themselves [58].In addition, Bohannon et al. evaluated the effect of the exposure to 6: FTS in white-footed mice exposed orally to 6:2 FTS for 112 days (4 weeks premating exposure plus at least 4 weeks mating exposure). The study highlighted the potential immunotoxicity of this compound but 6:2 FTS dosing did not alter sex steroid or thyroid hormone levels, thereby not supporting the shreds of evidence of altered serum hormone levels [8]. Taken together the available data would not support the presence of thyroid-related adverse effects for 6:2 FTS.

## PFAS mixtures, the real problem

Human health risk assessment of PFAS is up to now largely based upon animal toxicity data, derived from a single substance exposure. However, this strategy appears to not adequately assess the risk related to exposure to a mixture of compounds. Few in vitro studies focusing on PFAS mixtures revealed that these compounds could exert additive or synergistic/antagonistic effects, that could depend upon mixture components, target species, and dose ratio [99]. Following, an overview is provided of the recent studies aimed at assessing the effects of a mixture of PFAS on thyroid function in humans.

Li et al. compared the levels of eight PFASs (PFOS, PFOA, PFHxS, PFPeA, PFPrA, PFBA, PFHxA PFBS), thyroid function parameters (TSH, FT4, FT3) and thyroid antibodies (TgAb, and TMAb) that were evaluated in serum of the general population of some provinces in southern China [86]. The findings highlighted that ∑8PFASs as well as PFOS were significantly and negatively correlated with the serum levels of FT3 and FT4, whereas they were positively correlated with the serum levels of TSH. By going into detail, among these ∑8 PFASs, PFPeA and PFHxA were significantly and positively correlated with TgAb and TMAb. In the hypothyroidism and hyperthyroidism group, the levels of FT3, and TSH were opposite associated with PFOS, PFOA, and PFHxS, suggesting that these PFAS could induce an increase in the negative feedback mechanisms of TH at the pituitary level [86].

In the recent study by Liu et al., both alternative and legacy PFAS were detected in serum samples from 161 adults from Laizhou Bay, a chemical production site located in Shandong province (Northern China). The presence of an association between PFAS mixture serum levels (eleven perfluoroalkyl carboxylates PFCAs, three perfluoroalkane sulfonic acids PFSAs, 4 m-PFOA, 5 m-PFOA, isoPFOA,1 m-PFOS, 4 m-PFOS, 5 m-PFOS, isoPFOS 6:2 Cl-PFESA, 8:2 Cl-PFESA, ADONA) and thyroid parameters (free T4, total T4, free T3, total T3, TSH, TPOAb, TGAb) was investigated to assess potential health risks. The analysis of the effects of a single PFAS showed no significant association between serum PFOA concentrations (identified as the most abundant PFAS in human serum) and serum TSH levels or any other biomarkers of thyroid function. Multiple regression analysis showed that higher exposure of PFNA, PFDA, and n-PFOS, were associated with a marginal increase in free T4. No association with thyroid autoimmunity (TPOAb, TGAb), was shown. However, when PFAS were considered as a mixture and associated with thyroid hormone profile, the results showed that co-exposure of n-PFOA, PFNA, PFDA, and PFOS induced synergistic effects on free T4, while the mixture of serum PFBA and 6:2Cl-PFESA showed a synergistic influence on total and free T3. These findings suggest that both legacy and alternative PFAS could disrupt thyroid function [87]. Moving to pre-natal studies interesting data were provided in the “Shanghai Birth Cohort study” indicating that maternal exposure to PFAS mixtures in early pregnancy was positively associated with FT4 levels while being negatively related to TSH levels in TPOAb-positive women [3]. Another study by the same Authors suggested that cord serum TSH levels were negatively associated with neonatal PFOS, PFNA, PFDA, PFUnDA, and PFDoA exposures, and cord serum PFDoA increased FT4 levels [2]. In agreement with this study, Guo et al. measured the concentrations of 12 PFAS and 7 thyroid function indicators, reporting that some PFAS increased T4, and FT4 levels in cord serum, which was paralleled by a decrease in TSH levels [69]. In pregnant women and neonates, from the Boston Project Viva Cohort, higher concentrations of a PFAS mixture, which included PFOA, PFOS, PFNA, PFHxS, Et-PFOSA-AcOH, and Me-PFOSA-AcOH, were associated with decreased maternal and fetal free T4 indices [106]. Further analyses revealed that PFOA, PFHxS, Me-PFOSA-AcOH, and 2-(N-ethyl perfluoro octane sulfonamide) acetic acid (Et-PFOSA-AcOH) were the main contributors to the observed decrease in free T4 indices. No association was reported between the PFAS mixture and maternal T4 or TSH levels, but higher PFOS level was associated with increased maternal T4 levels, while increased PFOA level was associated with lower maternal free T4 index. PFHxS levels were non-linearly associated with maternal circulating TSH. Increased PFHxS levels were associated with decreased T4 levels in neonates as well [106]. Crute et al. [32] investigated the effects on maternal, fetal, and placental outcomes after preconceptional and gestational exposure to a PFAS mixture in a White rabbit model of New Zealand. This mixture contained 10 different perfluoroalkyl acids (PFAAs,PFHxA, PFPeA, PFHpA, PFBA, PFOA, PFOS, PFHxS, PFDA, PFNA, and PFBS) for a concentration of ΣPFAS near to 760 ng/L. Placental macroscopic changes were present in PFAS-exposed dams. In addition, lower circulating T4 and a non-significant trend for a higher T3:T4 ratio were found in these Dams. Goodrich et al., evaluated associations of PFAS mixtures (PFOS, PFHxS, PFHpS, PFOA, PFNA, and PFDA) with changes in metabolic pathways in an independent cohort of 137 young adults from the Southern California Children's Health Study (CHS) reporting a correlation with increased T4 levels [66].Wang et al. evaluated the associations of prenatal exposure to a mixture of 12 PFAS (PFOA, PFOS, PFHpA, PFNA, PFDA, PFHxS, PFUnDA, PFBS, PFDoA, PFHpS, PFDS, and PFOSA) with birth size to assess whether reproductive hormones and THs could have a role in these associations. In this view THs and PFAS levels were assessed in the cord serum of 1087 mother-newborn pairs obtained from the Sheyang Mini Birth Cohort Study, showing that PFNA exposure was positively associated with weight and that TSH appeared to mediate the associations between PFAS mixtures exposure and increased Ponderal index (PI) in high-dimensional mediation analysis framework [127]. Moreover, Cao et al., measured several PFAS (including 8:2 Cl-PFESA) and thyroid hormone levels in a birth cohort study of 1015 pairs of mothers and newborns in Wuhan, (China). PFASs and TSH concentration were quantified in cord blood sera. A decreased TSH concentration in all newborns was associated in a significant manner. In particular, it was observed that PFBS, PFOS, and 6:2 Cl-PFESA (56.50%, 18.71%, and 12.81%, respectively) were the greater influencers of the TSH decrease in neonates with the weights of. Interestingly, a negative association between cord serum PFBS and 8:2 Cl-PFESA with TSH concentration was observed in newborns, especially for boys[98]. Multiple linear regression models and Bayesian kernel machine regression models showed that prenatal PFAS mixture exposure, was positively associated with birth size and that such associations were partly mediated by cord serum TSH levels [15].

A recent study analyzed the correlation between prediagnostic serum levels of 19 PFAS and papillary thyroid cancer, showing a positive correlation between PFAS and the risk of developing thyroid cancer [92]. In addition, the ecological study by Alsen et al. examined the possible correlation between exposure to PFASs in drinking water and thyroid cancer incidence in the United States, finding a significant correlation between PFOA, PFNA and thyroid cancer, while no statistically significant correlation was found between PFOS, PFHxS, PFHpA or PFBS and thyroid cancer incidence.” [4].

Finally, Zang et al. enrolled a total of 295 gestational diabetes mellitus GDM cases and 295 controls from a prospective cohort of 2700 pregnant women in Shanghai. In total, 16 PFAS (PFBA, PFPeA, PFHxA, PFHpA, PFOA, PFNA, PFDA, PFUnDA, PFDoDA, PFBS, PFPeS, PFHxS, PFHpS, PFOS, 6:2 PFESA, and 8:2 PFESA) were measured in maternal spot serum samples in early pregnancy [131] to evaluate potential associations with circulating thyroid hormones. Several statistical regression models were applied in the analysis of PFAS exposure on the test for oral glucose tolerance and gestational diabetes mellitus (GDM) risk. It was found that PFOA exposure in pregnant women was associated with a higher risk of GDM, being this association consistent with elevated concentrations in GDM subjects as compared with controls. Mediation analysis showed an increase in the FT3 to FT4 ratio partially explaining the mechanism of this association [131].

## Conclusion

The present review article overviewed the available information regarding the currently most employed PFOA and PFOS alternatives and their potential thyroid adverse effects. The available data suggest that these alternatives, or at least some of them, should not be regarded as safe. Indeed, most of them were shown to produce thyroid interference at several levels. The thyroid-disrupting effects were recognized in vitro, in several animal models as well as in vivo* studies* in humans. The comparison between novel PFAS and their old predecessors highlights that the spectrum of adverse effects is not superimposable showing a sort of compound-related specificity. To add complexity, the effect of a single compound versus a mixture of compounds is not always easy to estimate. For both the above concepts, it appears clear that exposure time, concentration of a given PFAS as well as the model and/or species used may sustain different results.

Taken together the reviewed shreds of evidence might raise the following question: Are we searching for safer compounds or are we looking for an easy, rapid, and inexpensive way to replace banned compounds?

At present, the question remains unanswered but, at least in some cases, it appears reasonable to think about a general trend leading to the "restyling” of legacy PFAS which results in the production of legal but far to be proven safe and environmentally sustainable compounds.

In our opinion, the issue is a challenging one and to be judged as solved two main lines should be followed:

1. Studies specifically designed to test the safety profile of any newly produced PFAS alternative should be strongly encouraged
2. Regulation by specific laws aimed at limiting the release of PFAS in the environment together with active interventions with the final goal of removing PFAS from the environment. This latter aspect is surely not easy to address, but still, it represents the final goal to be achieved.

In this view, several technical approaches are ongoing to remove PFAS from the waters. On the other hand, if we continue introducing PFAS in the environment, this will become a “never-ending story”.

### Supplementary Information

Below is the link to the electronic supplementary material.

Supplementary file1 (DOCX 15 KB)

## Data Availability

Not applicable.

## References

[1] Agency LBE (2016) PFOA Und ADONA Messungen an Der Probenahmestelle Alz

[2] Aimuzi R, Luo K, Chen Q, Wang H, Feng L, Ouyang F, Zhang J (2019) Perfluoroalkyl and polyfluoroalkyl substances and fetal thyroid hormone levels in umbilical cord blood among newborns by prelabor caesarean delivery. Environ Int 130:104929 31228788 10.1016/j.envint.2019.104929 PMC7021220

[3] Aimuzi R, Luo K, Huang R, Huo X, Nian M, Ouyang F, Du Y, Feng L, Wang W, Zhang J et al (2020) Perfluoroalkyl and polyfluroalkyl substances and maternal thyroid hormones in early pregnancy. Environ Pollut 264:114557 32388293 10.1016/j.envpol.2020.114557

[4] Alsen M, Leung AM, van Gerwen M (2023) Per- and polyfluoroalkyl substances (PFAS) in community water systems (CWS) and the risk of thyroid cancer: an ecological study. Toxics 11:786 37755796 10.3390/toxics11090786 PMC10537801

[5] ARPAV (2019) Il composto cC6O4 nel Po: i monitoraggi effettuati al 23 luglio 2019

[6] Barry V, Winquist A, Steenland K (2013) Perfluorooctanoic acid (PFOA) exposures and incident cancers among adults living near a chemical plant. Environ Health Perspect 121:1313–1318 24007715 10.1289/ehp.1306615 PMC3855514

[7] Behnisch PA, Besselink H, Weber R, Willand W, Huang J, Brouwer A (2021) Developing potency factors for thyroid hormone disruption by PFASs using TTR-TRβ CALUX® bioassay and assessment of PFASs mixtures in technical products. Environ Int 157:106791 34364217 10.1016/j.envint.2021.106791

[8] Bohannon ME, Narizzano AM, Guigni BA, East AG, Quinn MJ (2023) Next-generation PFAS 6:2 fluorotelomer sulfonate reduces plaque formation in exposed white-footed mice. Toxicol Sci 192:97–105 36629485 10.1093/toxsci/kfad006

[9] Brandsma SH, Koekkoek JC, van Velzen MJM, de Boer J (2019) The PFOA substitute GenX detected in the environment near a fluoropolymer manufacturing plant in the Netherlands. Chemosphere 220:493–500 30594801 10.1016/j.chemosphere.2018.12.135

[10] Brendel S, Fetter É, Staude C, Vierke L, Biegel-Engler A (2018) Short-chain perfluoroalkyl acids: environmental concerns and a regulatory strategy under REACH. Environ Sci Eur 30:9 29527446 10.1186/s12302-018-0134-4 PMC5834591

[11] Brunn H, Arnold G, Körner W, Rippen G, Steinhäuser KG, Valentin I (2023) PFAS: forever chemicals—persistent, bioaccumulative and mobile. Reviewing the status and the need for their phase out and remediation of contaminated sites. Environ Sci Eur 35:20 10.1186/s12302-023-00721-8

[12] C H (2019) The hunt is on for GenX chemicals in people: analysis of North Carolina residents’ blood for Chemours PFAS yields surprises. Chemical & Engineering News

[13] California B (2016) Results for chemical groups. Cancer IAfRo IARC Monographs evaluate the carcinogenicity of perfluorooctanoic acid (PFOA) and perfluorooctanesulfonic acid (PFOS). IARC

[14] Cao XY, Liu J, Zhang YJ, Wang Y, Xiong JW, Wu J, Chen L (2020) Exposure of adult mice to perfluorobutanesulfonate impacts ovarian functions through hypothyroxinemia leading to down-regulation of Akt-mTOR signaling. Chemosphere 244:125497 31809938 10.1016/j.chemosphere.2019.125497

[15] Cao Z, Li J, Yang M, Gong H, Xiang F, Zheng H, Cai X, Xu S, Zhou A, Xiao H (2023) Prenatal exposure to perfluorooctane sulfonate alternatives and associations with neonatal thyroid stimulating hormone concentration: a birth cohort study. Chemosphere 311:136940 36273603 10.1016/j.chemosphere.2022.136940

[16] Cathey AL, Nguyen VK, Colacino JA, Woodruff TJ, Reynolds P, Aung MT (2023) Exploratory profiles of phenols, parabens, and per- and poly-fluoroalkyl substances among NHANES study participants in association with previous cancer diagnoses. J Expo Sci Environ Epidemiol 33:687–698 37718377 10.1038/s41370-023-00601-6 PMC10541322

[17] COMMISSION E (2020) COMMISSION STAFF WORKING DOCUMENT. Poly- and perfluoroalkyl substances (PFAS). Chemicals strategy for sustainability towards a toxic-free environment.

[18] COMMISSION E (2022) Proposal for a Directive amending the Water Framework Directive, the Groundwater Directive and the Environmental Quality Standards Directive. ENVIRONMENT.

[19] COMMISSION TE (2020) COMMISSION DELEGATED REGULATION (EU) 2020/1203 of 9 June 2020 amending Annex I to Regulation (EU) 2019/1021 of the European Parliament and of the Council as regards the entry for perfluorooctane sulfonic acid and its derivatives (PFOS). Official Journal of the European Union, pp 1–3

[20] COMMISSION TE (2022) COMMISSION REGULATION (EU) 2022/2388 of 7 December 2022 amending Regulation (EC) No 1881/2006 as regards maximum levels of perfluoroalkyl substances in certain foodstuffs. Official Journal of the European Union.

[21] COMMISSION TE (2022) COMMISSION RECOMMENDATION (EU) 2022/1431 of 24 August 2022 on the monitoring of perfluoroalkyl substances in food. Official Journal of the European Union

[22] COMMISSION TE (2022d) Commission Implementing Regulation (EU) 2022/1428 of 24 August 2022 laying down methods of sampling and analysis for the control of perfluoroalkyl substances in certain foodstuffs (Text with EEA relevance)

[23] Concepción-Zavaleta MJ, Coronado-Arroyo JC, Quiroz-Aldave JE, Concepción-Urteaga LA, Paz-Ibarra J (2023) Thyroid dysfunction and female infertility. A comprehensive review. Diabetes Metab Syndr 17:102876 37866272 10.1016/j.dsx.2023.102876

[24] Conder J, Zodrow J, Arblaster J, Kelly B, Gobas F, Suski J, Osborn E, Frenchmeyer M, Divine C, Leeson A (2021) Strategic resources for assessing PFAS ecological risks at AFFF sites. Integr Environ Assess Manag 17:746–752 33751777 10.1002/ieam.4405

[25] Conley JM, Lambright CS, Evans N, McCord J, Strynar MJ, Hill D, Medlock-Kakaley E, Wilson VS, Gray LE (2021) Hexafluoropropylene oxide-dimer acid (HFPO-DA or GenX) alters maternal and fetal glucose and lipid metabolism and produces neonatal mortality, low birthweight, and hepatomegaly in the Sprague-Dawley rat. Environ Int 146:106204 33126064 10.1016/j.envint.2020.106204 PMC7775906

[26] Coperchini F, Awwad O, Rotondi M, Santini F, Imbriani M, Chiovato L (2017) Thyroid disruption by perfluorooctane sulfonate (PFOS) and perfluorooctanoate (PFOA). J Endocrinol Investig 40:105–121 27837466 10.1007/s40618-016-0572-z

[27] Coperchini F, Croce L, Ricci G, Magri F, Rotondi M, Imbriani M, Chiovato L (2020) Thyroid-disrupting effects of old and new generation PFAS. Front Endocrinol (Lausanne) 11:612320 33542707 10.3389/fendo.2020.612320 PMC7851056

[28] Coperchini F, Croce L, Denegri M, Pignatti P, Agozzino M, Netti GS, Imbriani M, Rotondi M, Chiovato L (2020) Adverse effects of in vitro GenX exposure on rat thyroid cell viability, DNA integrity and thyroid-related genes expression. Environ Pollut 264:114778 32417585 10.1016/j.envpol.2020.114778

[29] Coperchini F, Croce L, Pignatti P, Ricci G, Gangemi D, Magri F, Imbriani M, Rotondi M, Chiovato L (2021) The new generation PFAS C6O4 does not produce adverse effects on thyroid cells in vitro. J Endocrinol Investig 44:1625–1635 33315184 10.1007/s40618-020-01466-4 PMC8285310

[30] Coperchini F, De Marco G, Croce L, Denegri M, Greco A, Magri F, Tonacchera M, Imbriani M, Rotondi M, Chiovato L (2023) PFOA, PFHxA and C6O4 differently modulate the expression of CXCL8 in normal thyroid cells and in thyroid cancer cell lines. Environ Sci Pollut Res Int 30:63522–63534 37052835 10.1007/s11356-023-26797-6

[31] Crofton KM (2008) Thyroid disrupting chemicals: mechanisms and mixtures. Int J Androl 31:209–223 18217984 10.1111/j.1365-2605.2007.00857.x

[32] Crute CE, Landon CD, Garner A, Hall SM, Everitt JI, Zhang S, Blake B, Olofsson D, Chen H, Stapleton HM et al (2023) Maternal exposure to perfluorobutane sulfonate (PFBS) during pregnancy: evidence of adverse maternal and fetoplacental effects in New Zealand White (NZW) rabbits. Toxicol Sci 191:239–252 36453863 10.1093/toxsci/kfac126 PMC9936209

[33] De Felice M, Di Lauro R (2004) Thyroid development and its disorders: genetics and molecular mechanisms. Endocr Rev 25:722–746 15466939 10.1210/er.2003-0028

[34] De Felice M, Di Lauro R (2011) Minireview: Intrinsic and extrinsic factors in thyroid gland development: an update. Endocrinology 152:2948–2956 21693675 10.1210/en.2011-0204

[35] De Silva AO, Spencer C, Ho KC, Al Tarhuni M, Go C, Houde M, de Solla SR, Lavoie RA, King LE, Muir DC et al (2016) Perfluoroalkylphosphinic acids in northern pike (*Esox lucius*), double-crested cormorants (*Phalacrocorax auritus*), and bottlenose dolphins (*Tursiops truncatus*) in relation to other perfluoroalkyl acids. Environ Sci Technol 50:10903–10913 27677975 10.1021/acs.est.6b03515

[36] De Toni L, Di Nisio A, Rocca MS, Pedrucci F, Garolla A, Dall’Acqua S, Guidolin D, Ferlin A, Foresta C (2022) Comparative evaluation of the effects of legacy and new generation perfluoralkyl substances (PFAS) on thyroid cells. Front Endocrinol (Lausanne) 13:915096 35813651 10.3389/fendo.2022.915096 PMC9259843

[37] Deng M, Wu Y, Xu C, Jin Y, He X, Wan J, Yu X, Rao H, Tu W (2018) Multiple approaches to assess the effects of F-53B, a Chinese PFOS alternative, on thyroid endocrine disruption at environmentally relevant concentrations. Sci Total Environ 624:215–224 29253770 10.1016/j.scitotenv.2017.12.101

[38] Dharpure R, Pramanik S, Pradhan A (2023) In silico analysis decodes transthyretin (TTR) binding and thyroid-disrupting effects of per- and polyfluoroalkyl substances (PFAS). Arch Toxicol 97:755–768 36566436 10.1007/s00204-022-03434-8 PMC9968702

[39] Dickman RA, Aga DS (2022) A review of recent studies on toxicity, sequestration, and degradation of per- and polyfluoroalkyl substances (PFAS). J Hazard Mater 436:129120 35643010 10.1016/j.jhazmat.2022.129120 PMC12981655

[40] Dixit F, Barbeau B, Mostafavi SG, Mohseni M (2020) Efficient removal of GenX (HFPO-DA) and other perfluorinated ether acids from drinking and recycled waters using anion exchange resins. J Hazard Mater 384:121261 31574386 10.1016/j.jhazmat.2019.121261

[41] Domingo JL (2012) Health risks of dietary exposure to perfluorinated compounds. Environ Int 40:187–195 21864910 10.1016/j.envint.2011.08.001

[42] Domingo JL, Nadal M (2017) Per- and polyfluoroalkyl substances (PFASs) in food and human dietary intake: a review of the recent scientific literature. J Agric Food Chem 65:533–543 28052194 10.1021/acs.jafc.6b04683

[43] Dupain C, Ali HM, Mouhoub TA, Urbinati G, Massaad-Massade L (2016) Induction of TTF-1 or PAX-8 expression on proliferation and tumorigenicity in thyroid carcinomas. Int J Oncol 49:1248–1258 27573549 10.3892/ijo.2016.3617

[44] DuPont (2017) DuPont-C30031_516655:determination of HFPO-DA in EDTA human plasma samples. Charles River Laboratories. The Chemours Company.U.S. EPA HEROID:4353920

[45] ECHA (2007) ammonium 2,2,3 trifluor-3-(1,1,2,2,3,3-hexafluoro-3-trifluormethoxypropoxy), propionate

[46] ECHA (2009) Difluoro{[2,2,4,5-tetrafluoro-5-(trifluoromethoxy)-1,3-dioxolan-4-yl]oxy}acetic acid.

[47] ECHA (2022) Long-chain perfluorocarboxylic acids (PFCAs), their salts and related compounds

[48] ECHA (2023) ECHA publishes PFAS restriction proposal ECHA/NR/23/04

[49] EFSA (2014) Scientific Opinion on the safety assessment of the substance, Perfluoro{acetic acid, 2-[(5-methoxy-1,3-ioxolan-4-yl)oxy]}, ammonium salt, CAS No 1190931-27-1, for use in food contact materials., p 3718. EFSA JOURNAL

[50] EFSA (2020) PFAS in food: EFSA assesses risks and sets tolerable intake

[51] Egalini F, Marinelli L, Rossi M, Motta G, Prencipe N, Rossetto Giaccherino R, Pagano L, Grottoli S, Giordano R (2022) Endocrine disrupting chemicals: effects on pituitary, thyroid and adrenal glands. Endocrine 78:395–405 35604630 10.1007/s12020-022-03076-x PMC9637063

[52] Ehsan MN, Riza M, Pervez MN, Khyum MMO, Liang Y, Naddeo V (2023) Environmental and health impacts of PFAS: sources, distribution and sustainable management in North Carolina (USA). Sci Total Environ 878:163123 37001657 10.1016/j.scitotenv.2023.163123

[53] EPA (2016) Basic information about per- and Polyfluoroalkyl substances (PFASs)

[54] EPA (2023) Our Current Understanding of the Human Health and Environmental Risks of PFAS

[55] EPA (2023) Documentation of changes made during OMB review under EO 12866 review—proposed PFAS National Primary Drinking Water Regulation

[56] EPA DMotE (2015) Short-chain polyfluoroalkyl substances (PFAS)

[57] Feng X, Cao X, Zhao S, Wang X, Hua X, Chen L (2017) Exposure of pregnant mice to perfluorobutanesulfonate causes hypothyroxinemia and developmental abnormalities in female offspring. Toxicol Sci 155:409–419 27803384 10.1093/toxsci/kfw219

[58] Flynn RW, Hoover G, Iacchetta M, Guffey S, de Perre C, Huerta B, Li W, Hoverman JT, Lee L, Sepúlveda MS (2022) comparative toxicity of aquatic per- and polyfluoroalkyl substance exposure in three species of amphibians. Environ Toxicol Chem 41:1407–1415 35199880 10.1002/etc.5319 PMC9314107

[59] Forrest D, Visser TJ (2013) Thyroid hormone signaling. Biochim Biophys Acta 1830:3859 23651664 10.1016/j.bbagen.2013.03.001

[60] Fromme H, Wöckner M, Roscher E, Völkel W (2017) ADONA and perfluoroalkylated substances in plasma samples of German blood donors living in South Germany. Int J Hyg Environ Health 220:455–460 28073630 10.1016/j.ijheh.2016.12.014

[61] Fry K, Power MC (2017) Persistent organic pollutants and mortality in the United States, NHANES 1999–2011. Environ Health 16:105 29017533 10.1186/s12940-017-0313-6 PMC5634885

[62] Gebbink WA, van Asseldonk L, van Leeuwen SPJ (2017) Presence of emerging per- and polyfluoroalkyl substances (PFASs) in river and drinking water near a fluorochemical production plant in the Netherlands. Environ Sci Technol 51:11057–11065 28853567 10.1021/acs.est.7b02488 PMC5677760

[63] Gebbink WA, Bossi R, Rigét FF, Rosing-Asvid A, Sonne C, Dietz R (2016) Observation of emerging per- and polyfluoroalkyl substances (PFASs) in Greenland marine mammals. Chemosphere 144:2384–2391 26610298 10.1016/j.chemosphere.2015.10.116

[64] Gilbert ME, O’Shaughnessy KL, Axelstad M (2020) Regulation of thyroid-disrupting chemicals to protect the developing brain. Endocrinology. 10.1210/endocr/bqaa106 32615585 10.1210/endocr/bqaa106 PMC8650774

[65] Girardi P, Rosina A, Merler E (2018) La concentrazione di sostanze perfluorurate nel sangue dei dipendenti ed ex dipendenti delle ditte RIMAR e MITENI (Trissino, Vicenza)

[66] Goodrich JA, Walker DI, He J, Lin X, Baumert BO, Hu X, Alderete TL, Chen Z, Valvi D, Fuentes ZC et al (2023) Metabolic signatures of youth exposure to mixtures of per- and polyfluoroalkyl substances: a multi-cohort study. Environ Health Perspect 131:27005 36821578 10.1289/EHP11372 PMC9945578

[67] Gordon SC (2011) Toxicological evaluation of ammonium 4,8-dioxa-3H-perfluorononanoate, a new emulsifier to replace ammonium perfluorooctanoate in fluoropolymer manufacturing. Regul Toxicol Pharmacol 59:64–80 20875479 10.1016/j.yrtph.2010.09.008

[68] Graceli JB, Dettogni RS, Merlo E, Niño O, da Costa CS, Zanol JF, Ríos Morris EA, Miranda-Alves L, Denicol AC (2020) The impact of endocrine-disrupting chemical exposure in the mammalian hypothalamic-pituitary axis. Mol Cell Endocrinol 518:110997 32841708 10.1016/j.mce.2020.110997

[69] Guo J, Zhang J, Wang Z, Zhang L, Qi X, Zhang Y, Chang X, Wu C, Zhou Z (2021) Umbilical cord serum perfluoroalkyl substance mixtures in relation to thyroid function of newborns: findings from Sheyang Mini Birth Cohort Study. Chemosphere 273:129664 33493812 10.1016/j.chemosphere.2021.129664

[70] Guo R, Reiner EJ, Bhavsar SP, Helm PA, Mabury SA, Braekevelt E, Tittlemier SA (2012) Determination of polyfluoroalkyl phosphoric acid diesters, perfluoroalkyl phosphonic acids, perfluoroalkyl phosphinic acids, perfluoroalkyl carboxylic acids, and perfluoroalkane sulfonic acids in lake trout from the Great Lakes region. Anal Bioanal Chem 404:2699–2709 22722738 10.1007/s00216-012-6125-1

[71] Harding-Marjanovic KC, Houtz EF, Yi S, Field JA, Sedlak DL, Alvarez-Cohen L (2015) Aerobic biotransformation of fluorotelomer thioether amido sulfonate (Lodyne) in AFFF-amended microcosms. Environ Sci Technol 49:7666–7674 26042823 10.1021/acs.est.5b01219

[72] Hernandez A, Martinez ME, Chaves C, Anselmo J (2023) Epigenetic developmental programming and intergenerational effects of thyroid hormones. Vitam Horm 122:23–49 36863795 10.1016/bs.vh.2023.01.003 PMC10938172

[73] Hong SH, Lee SH, Yang JY, Lee JH, Jung KK, Seok JH, Kim SH, Nam KT, Jeong J, Lee JK et al (2020) Orally administered 6:2 chlorinated polyfluorinated ether sulfonate (F-53B) causes thyroid dysfunction in rats. Toxics 8:54 32784452 10.3390/toxics8030054 PMC7560397

[74] Hopkins ZR, Sun M, DeWitt JC, Knappe DRU (2018) Recently detected drinking water contaminants: GenX and other per- and polyfluoroalkyl ether acids. American Water Works Association

[75] Howard PH, Muir DC (2010) Identifying new persistent and bioaccumulative organics among chemicals in commerce. Environ Sci Technol 44:2277–2285 20163179 10.1021/es903383a

[76] Inspectorate TDW (2020) PFAS and forever chemicals

[77] Kim S, Stroski KM, Killeen G, Smitherman C, Simcik MF, Brooks BW (2020) 8:8 Perfluoroalkyl phosphinic acid affects neurobehavioral development, thyroid disruption, and DNA methylation in developing zebrafish. Sci Total Environ 736:139600 32474277 10.1016/j.scitotenv.2020.139600

[78] Kleinau G, Neumann S, Grüters A, Krude H, Biebermann H (2013) Novel insights on thyroid-stimulating hormone receptor signal transduction. Endocr Rev 34:691–724 23645907 10.1210/er.2012-1072 PMC3785642

[79] Kotlarz N, McCord J, Collier D, Lea CS, Strynar M, Lindstrom AB, Wilkie AA, Islam JY, Matney K, Tarte P et al (2020) Measurement of novel, drinking water-associated PFAS in blood from adults and children in Wilmington, North Carolina. Environ Health Perspect 128:77005 32697103 10.1289/EHP6837 PMC7375159

[80] Kudo N, Suzuki-Nakajima E, Mitsumoto A, Kawashima Y (2006) Responses of the liver to perfluorinated fatty acids with different carbon chain length in male and female mice: in relation to induction of hepatomegaly, peroxisomal beta-oxidation and microsomal 1-acylglycerophosphocholine acyltransferase. Biol Pharm Bull 29:1952–1957 16946516 10.1248/bpb.29.1952

[81] Kudo N, Suzuki E, Katakura M, Ohmori K, Noshiro R, Kawashima Y (2001) Comparison of the elimination between perfluorinated fatty acids with different carbon chain length in rats. Chem Biol Interact 134:203–216 11311214 10.1016/S0009-2797(01)00155-7

[82] Lee H, Mabury SA (2011) A pilot survey of legacy and current commercial fluorinated chemicals in human sera from United States donors in 2009. Environ Sci Technol 45:8067–8074 21486041 10.1021/es200167q

[83] Lee H, Mabury SA (2017) Sorption of perfluoroalkyl phosphonates and perfluoroalkyl phosphinates in soils. Environ Sci Technol 51:3197–3205 28222593 10.1021/acs.est.6b04395

[84] Lee SY, Pearce EN (2023) Hyperthyroidism: a review. JAMA 330:1472–1483 37847271 10.1001/jama.2023.19052 PMC10873132

[85] Li H, Yang M, Yang J, Seery S, Ma C, Liu Y, Zhang X, Li A, Guo H (2023) Per- and polyfluoroalkyl substances and the associated thyroid cancer risk: a case-control study in China. Chemosphere 337:139411 37419160 10.1016/j.chemosphere.2023.139411

[86] Li Y, Cheng Y, Xie Z, Zeng F (2017) Perfluorinated alkyl substances in serum of the southern Chinese general population and potential impact on thyroid hormones. Sci Rep 7:43380 28240244 10.1038/srep43380 PMC5327476

[87] Liu J, Song L, Zhan J, Zhong Y, Shi Z (2023) Occurrence of legacy and alternative per- and polyfluoroalkyl substances in serum from high exposure population and their disrupting effects on serum lipids and thyroid function. Sci Total Environ 878:162988 36958558 10.1016/j.scitotenv.2023.162988

[88] Liu J, Gao X, Wang Y, Leng J, Li J, Zhao Y, Wu Y (2021) Profiling of emerging and legacy per-/polyfluoroalkyl substances in serum among pregnant women in China. Environ Pollut 271:116376 33383424 10.1016/j.envpol.2020.116376

[89] Liu M, Yi S, Chen P, Chen M, Zhong W, Yang J, Sun B, Zhu L (2019) Thyroid endocrine disruption effects of perfluoroalkyl phosphinic acids on zebrafish at early development. Sci Total Environ 676:290–297 31048160 10.1016/j.scitotenv.2019.04.177

[90] Liu M, Zhang G, Meng L, Han X, Li Y, Shi Y, Li A, Turyk ME, Zhang Q, Jiang G (2022) Associations between novel and legacy per- and polyfluoroalkyl substances in human serum and thyroid cancer: a case and healthy population in Shandong Province, East China. Environ Sci Technol 56:6144–6151 34618433 10.1021/acs.est.1c02850

[91] Liu W, Yang J, Li J, Zhang J, Zhao J, Yu D, Xu Y, He X, Zhang X (2020) Toxicokinetics and persistent thyroid hormone disrupting effects of chronic developmental exposure to chlorinated polyfluorinated ether sulfonate in Chinese rare minnow. Environ Pollut 263:114491 32304979 10.1016/j.envpol.2020.114491

[92] Madrigal JM, Troisi R, Surcel HM, Öhman H, Kivelä J, Kiviranta H, Rantakokko P, Koponen J, Medgyesi DN, Kitahara CM et al (2024) Prediagnostic serum concentrations of per- and polyfluoroalkyl substances and risk of papillary thyroid cancer in the Finnish Maternity Cohort. Int J Cancer 154:979–991 37902275 10.1002/ijc.34776 PMC11286200

[93] Messmer MF, Salloway J, Shara N, Locwin B, Harvey MW, Traviss N (2022) Risk of cancer in a community exposed to per- and poly-fluoroalkyl substances. Environ Health Insights 16:11786302221076708 35173445 10.1177/11786302221076707 PMC8842173

[94] National Toxicology Program Public Health Service USDoHaHS (2019) NTP TOX 96: NTP Technical Report on the toxicity studies of perfluoroalkyl sulfonates (perfluorobutane sulfonic acid, perfluorohexane sulfonate potassium salt, and perfluorooctane sulfonic acid) administered by gavage to sprague dawley (Hsd: Sprague Dawley SD) rats. pp ISSN 2378-8992 National Toxicology Program Public Health Service, U.S. Department of Health and Human Services: Research Triangle Park

[95] National Toxicology Program Public Health Service USDoHaHS (2019) NTPTOX97: NTP Technical Report on the toxicity studies of perfluoroalkyl carboxylates (perfluorohexanoic acid, perfluorooctanoic acid, perfluorononanoic acid, and perfluorodecanoic acid) administered by gavage to sprague dawley (Hsd: Sprague Dawley SD) Rats. pp ISSN 2378-8992: National Toxicology Program Public Health Service, U.S. Department of Health and Human Services: Research Triangle Park

[96] OECD (2018) OECD guideline for the testing of chemicals

[97] OECD (2019) Drinking water. environment

[98] OECD (2023) Substances of very high concern under REACH

[99] Ojo AF, Peng C, Ng JC (2021) Assessing the human health risks of per- and polyfluoroalkyl substances: a need for greater focus on their interactions as mixtures. J Hazard Mater 407:124863 33373965 10.1016/j.jhazmat.2020.124863

[100] Oliveira KJ, Chiamolera MI, Giannocco G, Pazos-Moura CC, Ortiga-Carvalho TM (2018) Thyroid function disruptors: from nature to chemicals. J Mol Endocrinol 62:R1–R19 10.1530/JME-18-0081 30006341

[101] Ortiga-Carvalho TM, Sidhaye AR, Wondisford FE (2014) Thyroid hormone receptors and resistance to thyroid hormone disorders. Nat Rev Endocrinol 10:582–591 25135573 10.1038/nrendo.2014.143 PMC4578869

[102] Ortiga-Carvalho TM, Chiamolera MI, Pazos-Moura CC, Wondisford FE (2016) Hypothalamus–pituitary–thyroid axis. Compr Physiol 6:1387–1428 27347897 10.1002/cphy.c150027

[103] Özel F, Rüegg J (2023) Exposure to endocrine-disrupting chemicals and implications for neurodevelopment. Dev Med Child Neurol 65:1005–1011 36808586 10.1111/dmcn.15551

[104] Pan Y, Zhang H, Cui Q, Sheng N, Yeung LWY, Guo Y, Sun Y, Dai J (2017) First report on the occurrence and bioaccumulation of hexafluoropropylene oxide trimer acid: an emerging concern. Environ Sci Technol 51:9553–9560 28780851 10.1021/acs.est.7b02259

[105] Pandelides Z, Conder J, Choi Y, Allmon E, Hoskins T, Lee L, Hoverman J, Sepúlveda M (2023) A critical review amphibian PFAS ecotoxicity research studies: identification of screening levels in water and other useful resources for site-specific ecological risk assessments. Environ Toxicol Chem 42:2078–2090 37314102 10.1002/etc.5695

[106] Preston EV, Webster TF, Claus Henn B, McClean MD, Gennings C, Oken E, Rifas-Shiman SL, Pearce EN, Calafat AM, Fleisch AF et al (2020) Prenatal exposure to per- and polyfluoroalkyl substances and maternal and neonatal thyroid function in the Project Viva Cohort: A mixtures approach. Environ Int 139:105728 32311629 10.1016/j.envint.2020.105728 PMC7282386

[107] Ruan T, Lin Y, Wang T, Liu R, Jiang G (2015) Identification of novel polyfluorinated ether sulfonates as PFOS alternatives in municipal sewage sludge in China. Environ Sci Technol 49:6519–6527 25961764 10.1021/acs.est.5b01010

[108] Shi G, Cui Q, Wang J, Guo H, Pan Y, Sheng N, Guo Y, Dai J (2019) Chronic exposure to 6:2 chlorinated polyfluorinated ether sulfonate acid (F-53B) induced hepatotoxic effects in adult zebrafish and disrupted the PPAR signaling pathway in their offspring. Environ Pollut 249:550–559 30928526 10.1016/j.envpol.2019.03.032

[109] Steenland K, Woskie S (2012) Cohort mortality study of workers exposed to perfluorooctanoic acid. Am J Epidemiol 176:909–917 23079607 10.1093/aje/kws171

[110] Steenland K, Fletcher T, Savitz DA (2010) Epidemiologic evidence on the health effects of perfluorooctanoic acid (PFOA). Environ Health Perspect 118:1100–1108 20423814 10.1289/ehp.0901827 PMC2920088

[111] Stoker TE, Wang J, Murr AS, Bailey JR, Buckalew AR (2023) High-throughput screening of ToxCast PFAS chemical library for potential inhibitors of the human sodium iodide symporter. Chem Res Toxicol 36:380–389 36821091 10.1021/acs.chemrestox.2c00339 PMC12050117

[112] Strynar M, Dagnino S, McMahen R, Liang S, Lindstrom A, Andersen E, McMillan L, Thurman M, Ferrer I, Ball C (2015) Identification of Novel perfluoroalkyl ether carboxylic acids (PFECAs) and sulfonic acids (PFESAs) in natural waters using accurate mass time-of-flight mass spectrometry (TOFMS). Environ Sci Technol 49:11622–11630 26392038 10.1021/acs.est.5b01215

[113] Sun B, Liu M, Tang L, Hu C, Huang Z, Chen L (2021) Probiotics inhibit the stunted growth defect of perfluorobutanesulfonate via stress and thyroid axes in zebrafish larvae. Environ Pollut 290:118013 34428700 10.1016/j.envpol.2021.118013

[114] Sun S, Guo H, Wang J, Dai J (2019) Hepatotoxicity of perfluorooctanoic acid and two emerging alternatives based on a 3D spheroid model. Environ Pollut 246:955–962 31159145 10.1016/j.envpol.2018.12.065

[115] Tang L, Liu M, Song S, Hu C, Lam PKS, Lam JCW, Chen L (2020) Interaction between hypoxia and perfluorobutane sulfonate on developmental toxicity and endocrine disruption in marine medaka embryos. Aquat Toxicol 222:105466 32172180 10.1016/j.aquatox.2020.105466

[116] Thambirajah AA, Wade MG, Verreault J, Buisine N, Alves VA, Langlois VS, Helbing CC (2022) Disruption by stealth—interference of endocrine disrupting chemicals on hormonal crosstalk with thyroid axis function in humans and other animals. Environ Res 203:111906 34418447 10.1016/j.envres.2021.111906

[117] Union CotE (2022) ANNEXES to the proposal for a Directive of the European Parliament and of the Council amending Directive 2000/60/EC establishing a framework for Community action in the field of water policy, Directive 2006/118/EC on the protection of groundwater against pollution and deterioration and Directive 2008/105/EC on environmental quality standards in the field of water policy

[118] UNION E (2020) Directive (EU) 2020/2184 of the European Parliament and of the Council of 16 December 2020 on the quality of water intended for human consumption (recast) (Text with EEA relevance)

[119] Union E (2020) amending Annex I to Regulation (EU) 2019/1021 of the European Parliament and of the Council as regards the entry for perfluorooctane sulfonic acid and its derivatives (PFOS). Official Journal of the European Union

[120] van Gerwen M, Colicino E, Guan H, Dolios G, Nadkarni GN, Vermeulen RCH, Wolff MS, Arora M, Genden EM, Petrick LM (2023) Per- and polyfluoroalkyl substances (PFAS) exposure and thyroid cancer risk. EBioMedicine 97:104831 37884429 10.1016/j.ebiom.2023.104831 PMC10667111

[121] Vieira VM, Hoffman K, Shin HM, Weinberg JM, Webster TF, Fletcher T (2013) Perfluorooctanoic acid exposure and cancer outcomes in a contaminated community: a geographic analysis. Environ Health Perspect 121:318–323 23308854 10.1289/ehp.1205829 PMC3621179

[122] Wagner A BT (2017) Chemours: GenX polluting the Cape Fear since 1980. StarNews Online

[123] Wang J, Shi G, Yao J, Sheng N, Cui R, Su Z, Guo Y, Dai J (2020) Perfluoropolyether carboxylic acids (novel alternatives to PFOA) impair zebrafish posterior swim bladder development via thyroid hormone disruption. Environ Int 134:105317 31733528 10.1016/j.envint.2019.105317

[124] Wang Q, Li P, Qi S, Yuan J, Ding Z (2023) Borderline personality disorder and thyroid diseases: a Mendelian randomization study. Front Endocrinol (Lausanne) 14:1259520 37854187 10.3389/fendo.2023.1259520 PMC10579900

[125] Wang S, Huang J, Yang Y, Hui Y, Ge Y, Larssen T, Yu G, Deng S, Wang B, Harman C (2013) First report of a Chinese PFOS alternative overlooked for 30 years: its toxicity, persistence, and presence in the environment. Environ Sci Technol 47:10163–10170 23952109 10.1021/es401525n

[126] Wang Z, Cousins IT, Scheringer M, Hungerbühler K (2013) Fluorinated alternatives to long-chain perfluoroalkyl carboxylic acids (PFCAs), perfluoroalkane sulfonic acids (PFSAs) and their potential precursors. Environ Int 60:242–248 24660230 10.1016/j.envint.2013.08.021

[127] Wang Z, Zhang J, Dai Y, Zhang L, Guo J, Xu S, Chang X, Wu C, Zhou Z (2023) Mediating effect of endocrine hormones on association between per- and polyfluoroalkyl substances exposure and birth size: findings from sheyang mini birth cohort study. Environ Res 226:115658 36894112 10.1016/j.envres.2023.115658

[128] Wen L, Shi YB (2015) Unliganded thyroid hormone receptor α controls developmental timing in Xenopus tropicalis. Endocrinology 156:721–734 25456066 10.1210/en.2014-1439 PMC4298314

[129] Xin Y, Ren XM, Ruan T, Li CH, Guo LH, Jiang G (2018) Chlorinated polyfluoroalkylether sulfonates exhibit similar binding potency and activity to thyroid hormone transport proteins and nuclear receptors as perfluorooctanesulfonate. Environ Sci Technol 52:9412–9418 30052437 10.1021/acs.est.8b01494

[130] Yin N, Yang R, Liang S, Hu B, Ruan T, Faiola F (2018) Evaluation of the early developmental neural toxicity of F-53B, as compared to PFOS, with an in vitro mouse stem cell differentiation model. Chemosphere 204:109–118 29655103 10.1016/j.chemosphere.2018.04.011

[131] Zang L, Liu X, Xie X, Zhou X, Pan Y, Dai J (2023) Exposure to per- and polyfluoroalkyl substances in early pregnancy, risk of gestational diabetes mellitus, potential pathways, and influencing factors in pregnant women: a nested case-control study. Environ Pollut 326:121504 36965679 10.1016/j.envpol.2023.121504

[132] Zhang S, Chen K, Li W, Chai Y, Zhu J, Chu B, Li N, Yan J, Yang Y (2021) Varied thyroid-disrupting effects of perfluorooctanoic acid (PFOA) and its novel alternatives hexafluoropropylene-oxide-dimer-acid (GenX) and ammonium 4,8-dioxa-3H-perfluorononanoate (ADONA) in vitro. Environ Int 156:106745 34246126 10.1016/j.envint.2021.106745

[133] Zhang YT, Zeeshan M, Su F, Qian ZM, Dee Geiger S, Edward McMillin S, Wang ZB, Dong PX, Ou YQ, Xiong SM et al (2022) Associations between both legacy and alternative per- and polyfluoroalkyl substances and glucose-homeostasis: the Isomers of C8 health project in China. Environ Int 158:106913 34624590 10.1016/j.envint.2021.106913

[134] Zhao L, Teng M, Zhao X, Li Y, Sun J, Zhao W, Ruan Y, Leung KMY, Wu F (2023) Insight into the binding model of per- and polyfluoroalkyl substances to proteins and membranes. Environ Int 175:107951 37126916 10.1016/j.envint.2023.107951

