# Supplementary material for: Changing the structure of PFOA and PFOS: a chemical industry strategy or a solution to avoid thyroid-disrupting effects?
Source: J Endocrinol Invest. 2024 Mar 24;47(8):1863–79. doi: 10.1007/s40618-024-02339-w (PMC11266260; doi:10.1007/s40618-024-02339-w)
Supplement: Supplementary file 1 — Supplementary file1 (DOCX 15 KB) [file 40618_2024_2339_MOESM1_ESM.docx]

**Supplemental material**

**Changing the structure of PFOA and PFOS: a chemical industry strategy or a solution to avoid thyroid disrupting effects?**

Francesca Coperchini^1^, Alessia Greco^1^, Mario Rotondi^1,2^

^1^ Department of Internal Medicine and Therapeutics, University of Pavia, 27100, Italy;

^2^ Istituti Clinici Scientifici Maugeri IRCCS, Unit of Endocrinology and Metabolism, Laboratory for Endocrine Disruptors, 27100, Pavia, Italy.

***Key words :*** *PFOA; PFOS; endocrine disruptor; thyroid*

***Short-title:*** *Novel-PFAS and thyroid disruption*

*Corresponding Author:*

Prof. Mario Rotondi, M.D., Ph.D.

Unit of Endocrinology and Metabolism, Istituti Clinici Scientifici Maugeri IRCCS

Department of Internal Medicine and Therapeutics, University of Pavia, Italy

Via S. Maugeri 4, I-27100, Pavia, Italy

Fax: +39-0382-592692

e-mail: [mario.rotondi@icsmaugeri.it](mailto:mario.rotondi@icsmaugeri.it)

**Table 1.** **PFAS - sum of 24”, expressed as PFOA equivalents , based on the potencies of the substances relative to that of PFOA**

| **Compound** | **Acronym** |
| --- | --- |
| Perfluorooctanoic acid | PFOA |
| Perfluorooctane sulfonic acid | PFOS |
| Perfluorohexane sulfonic acid | PFHxS |
| Perfluorononanoic acid | PFNA |
| Perfluorobutane sulfonic acid | PFBS |
| Perfluorohexanoic acid | PFHxA |
| Perfluorobutanoic acid | PFBA |
| Perfluoropentanoic acid | PFPeA |
| Perfluoropentane sulfonic acid | PFPeS |
| Perfluorodecanoic acid | PFDA |
| Perfluorododecanoic acid | PFDoDA |
| Perfluoroundecanoic acid | PFUnDA |
| Perfluoroheptanoic acid | PFHpA |
| Perfluorotridecanoic acid | PFTrDA |
| Perfluoroheptane sulfonic acid | PFHpS |
| Perfluorodecane sulfonic acid | PFDS |
| Perfluorotetradecanoic acid | PFTeDA |
| Perfluorohexadecanoic acid | PFHxDA |
| Perfluorooctadecanoic acid | PFODA |
| Ammonium perfluoro (2-methyl-3-oxahexanoate) | Gen X |
| Propanoic Acid/Ammonium 2,2,3-trifluoro-3-(1,1,2,2,3,3-  hexafluoro-3-(trifluoromethoxy) propoxy)propanoate | ADONA |
| 2-(Perfluorohexyl)ethyl alcohol | 6:2 FTOH |
| 2-(Perfluorooctyl)ethanol | 8:2 FTOH |
